# A High Throughput Genotyping Approach Reveals Distinctive Autosomal Genetic Signatures for European and Near Eastern Wild Boar

**DOI:** 10.1371/journal.pone.0055891

**Published:** 2013-02-27

**Authors:** Arianna Manunza, Ali Zidi, Seryozha Yeghoyan, Valentin Adrian Balteanu, Teodora Crina Carsai, Oleg Scherbakov, Oscar Ramírez, Shahin Eghbalsaied, Anna Castelló, Anna Mercadé, Marcel Amills

**Affiliations:** 1 Department of Animal Genetics, Center for Research in Agricultural Genomics (CSIC-IRTA-UAB-UB), Universitat Autònoma de Barcelona, Bellaterra, Spain; 2 Armenian State Agrarian University, Yerevan, Armenia; 3 Faculty of Animal Husbandry and Biotechnology, Department of Biotechnology, Regional Laboratory for Animals Genotyping, University of Agricultural Sciences and Veterinary Medicine Cluj-Napoca, Cluj County, Romania; 4 Institut de Biologia Evolutiva (Universitat Pompeu Fabra-CSIC), CEXS-UPF-PRBB, Barcelona, Spain; 5 Department of Animal Science, Khorasgan (Isfahan) branch, Islamic Azad University, Isfahan, Iran; 6 Departament de Ciència Animal i dels Aliments, Universitat Autònoma de Barcelona, Bellaterra, Spain; Ecole Normale Supérieure de Lyon, France

## Abstract

The lack of a Near Eastern genetic signature in modern European porcine breeds indicates that, although domestic pigs from the Fertile Crescent entered Europe during the Neolithic, they were completely replaced by their European counterparts in a short window of time. Whilst the absence of such genetic signature has been convincingly demonstrated at the mitochondrial level, variation at the autosomal genomes of European and Near Eastern *Sus scrofa* has not been compared yet. Herewith, we have explored the genetic relationships among 43 wild boar from Europe (N = 21), Near East (N = 19) and Korea (N = 3), and 40 Iberian (N = 16), Canarian (N = 4) and Mangalitza (N = 20) pigs by using a high throughput SNP genotyping platform. After data filtering, 37,167 autosomal SNPs were used to perform population genetics analyses. A multidimensional scaling plot based on genome-wide identity-by-state pairwise distances inferred with PLINK showed that Near Eastern and European wild boar populations are genetically differentiated. Maximum likelihood trees built with TreeMix supported this conclusion *i.e.* an early population split between Near Eastern and European *Sus scrofa* was observed. Moreover, analysis of the data with Structure evidenced that the sampled Iberian, Canarian and Mangalitza pigs did not carry any autosomal signature compatible with a Near Eastern ancestry, a finding that agrees well with previous mitochondrial studies.

## Introduction

A fundamental contribution to understand how pigs were domesticated was provided through the analysis of mitochondrial sequences from a worldwide sample of pigs and wild boar [1], [2]. This approach revealed, amongst other findings, that modern European pig breeds do not harbour Near Eastern mitochondrial haplotypes, suggesting that they descend from wild boar domesticated locally. In a subsequent study, the entry of Near Eastern pigs into Europe during the Neolithic was confirmed by identifying Near Eastern mitochondrial haplotypes in ancient pig samples from Romania, Germany and France [3]. This event was followed by the rapid replacement of Near Eastern domestic pigs by locally domesticated European swine (European haplotypes increased from 5% to 95% in a few hundred years), thus explaining the absence of a Near Eastern genetic signature in the mitochondrial gene pool of modern European breeds.

One important drawback of mitochondrial analyses is that they just give a partial view of the total diversity of a given species. As stated by Bruford [4], the mitochondrial genome is a limited predictor of overall genomic diversity, because it behaves like a single locus and is an extra-nuclear genetic marker with specific evolutionary dynamics. Given its maternal inheritance, mitochondrial DNA does not allow to detect paternal gene flow, which has a strong effect on the evolution of domestic species [4]. In an effort to overcome these limitations, we analysed a worldwide sample of *Sus scrofa* with a combination of mitochondrial, Y-chromosome and autosomal microsatellite markers [5]. Whilst we found that European and Near Eastern mitochondrial sequences clustered independently, in close agreement with Larson *et al.* [1], Y-chromosome and microsatellite allele frequencies were quite similar in both populations. On the basis of these findings, the presence of Near Eastern alleles in the autosomal gene pool of current European breeds could not be ascertained [5]. Herewith, we have investigated the relationships between European and Near Eastern wild boar plus three European pig breeds (Iberian, Canarian and Mangalitza) by employing the Illumina Porcine SNP60 BeadChip. This tool allows the simultaneous genotyping of 62,163 single nucleotide polymorphisms (SNPs) uniformly distributed in the pig genome [6], thus providing a level of unprecedented resolution in the framework of population genetic studies.

## Results and Discussion

By using a whole genome SNPs typing technique, we have analysed the autosomal diversity of wild boar from Europe (Spain, Belgium and Russia) and the Near East (Iran, Turkey and Armenia) as well as of domestic pigs from Spain (Iberian and Canarian breeds) and Romania (Mangalitza breed). Remarkably, the Romanian and Spanish populations are located at the two Eastern and Western extremes of the geographic distribution of European pig breeds, respectively. Besides, Iberian and Mangalitza pigs have not been significantly introgressed with Far Eastern blood [5]. A few wild boar from Korea were also included in the analysis as an “outgroup” (Western and Far Eastern *Sus scrofa* diverged about 0.6–1.6 MYR [7], [8], [9]. After quality control with the PLINK toolset [10], a total of 37,167 SNPs were selected to carry out genetic analyses. Expected and observed heterozygosities of pig and wild boar populations did not differ significantly, as shown in **Table 1**. Both parameters displayed values that are in the lower range of what has been reported so far. In this sense, Zhang and Plastow [11] described values of 0.54 (range: 0.35–0.65) and 0.57 (range: 0.35–0.71) for observed and expected heterozygosities in European pig populations genotyped with the Illumina Porcine SNP60 BeadChip. The low heterozygosity values we have observed in Iberian pigs and European and Near Eastern wild boar cannot be explained in terms of limited sampling (**Table S1**). One possible reason for this result would be ascertainment bias *i.e.* the Illumina Porcine SNP60 BeadChip was built on the basis of 19 reduced representation libraries derived from four swine breeds (Duroc, Landrace, Large White, Piétrain) and a single wild boar population [6], so the diversity of other unrelated *Sus scrofa* populations (*e.g.* Near Eastern wild boar, Iberian and Mangalitza pigs, etc.) can be severely underestimated. Of course, reduced diversity could be also the consequence of genetic drift combined with past demographic events such as founder effects and bottlenecks. In the case of wild boar, it is well known that excessive hunting and progressive loss of habitat have caused a sustained demographic decline that, in certain cases (*e.g.* United Kingdom), ended with the local extinction of this species [12]. Similarly, the census of the Iberian breed has also suffered a dramatic reduction since 1960 as a consequence of African swine fever outbreaks and competition with more productive foreign breeds [13].

**Table 1:** Observed and expected heterozygosities of Near Eastern (NEWB) and European (EUWB) wild boar and Iberian (IB) and Mangalitza (MA) pigs^1^.

Populations	Ho	He	*P*-value
**NEWB**	0.229	0.241	0.752
**MA**	0.358	0.314	0.705
**IB**	0.285	0.226	0.929
**EUWB**	0.264	0.292	0.705

1 Far Eastern wild boar and Canarian pigs were not included in this analysis because of insufficient sample size.

**Figure 1:**
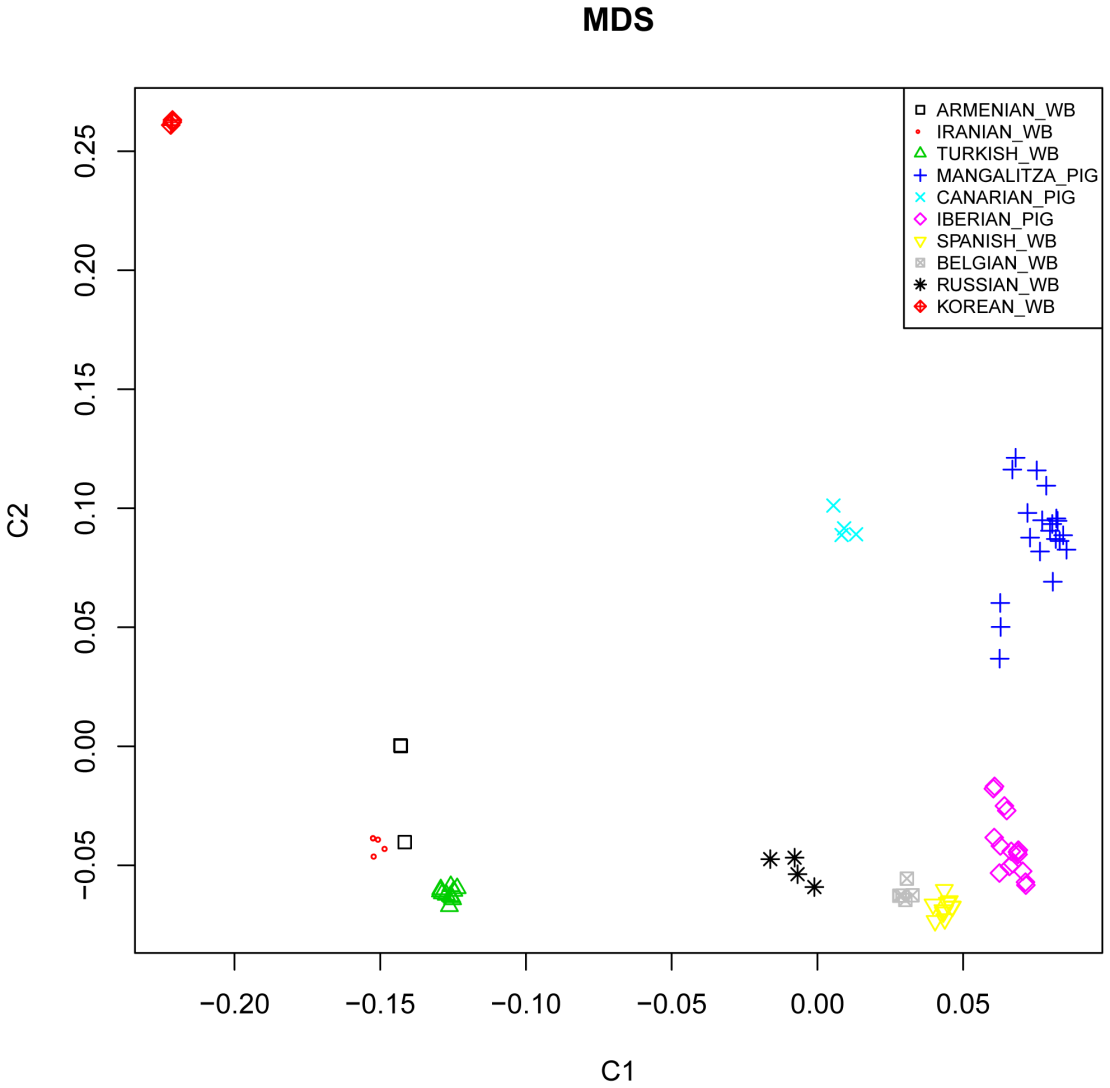
Multidimensional scaling plot based on genome-wide identity-by-state pairwise distances inferred with PLINK. This graph displays the genetic relationships between Near Eastern, Korean and European wild boar and Iberian, Canarian and Mangalitza pigs.

Our genetic analysis was performed to compare variation at the autosomal genomes of Near Eastern and European wild boar as well as three populations of European domestic pigs. The results obtained allow us to state that modern European and Near Eastern wild boar harbour clearly distinctive autosomal signatures. In this way, a multidimensional scaling plot based on genome-wide identity-by-state pairwise distances calculated with PLINK (**Figure 1** **, Figure S1**) showed that wild boar from Turkey, Iran and Armenia cluster together and independently from those of Russia, Belgium and Spain. In the study of Ramírez *et al.* [5], the level of differentiation between these two populations appeared to be less pronounced. This discrepancy is probably due to the fact that the Illumina Porcine SNP60 BeadChip has a much finer resolution than the microsatellite panel employed by these authors [5]. Moreover, none of the Iberian, Mangalitza and Canarian pigs grouped with the Near Eastern wild boar demonstrating that their current gene pools are fundamentally European. In summary, results shown in **Figure 1** and **Figure S1** illustrate that pig and wild boar specimens clustered in strict accordance with their geographic origin, a feature that evidences the existence of a significant level of population structure (even at a regional scale). This conclusion is supported by the highly significant pairwise F_ST_ values we have found, that range from 0.129 to 0.247 (**Table 2**), and the analysis of the molecular variance [14] that evidenced that 23.56% of the autosomal variation was contained in the among population component. These findings agree well with previous microsatellite studies revealing some level of population structure in pig breeds from Europe [15], [16] and Far East [17], [18].

**Table 2:** Pairwise F_ST_-values between Near Eastern (NEWB) and European (EUWB) wild boar and Iberian (IB) and Mangalitza pigs (MA)^1^^,^ 2.

Populations	NEWB	MA	IB
**MA**	0.244***	-	-
**IB**	0.247***	0.146***	-
**EUWB**	0.220***	0.164***	0.129***

1 Far Eastern wild boar and Canarian pigs were not included in this analysis because of insufficient sample size.

2 *** *P*-value<0.001.

**Figure 2:**
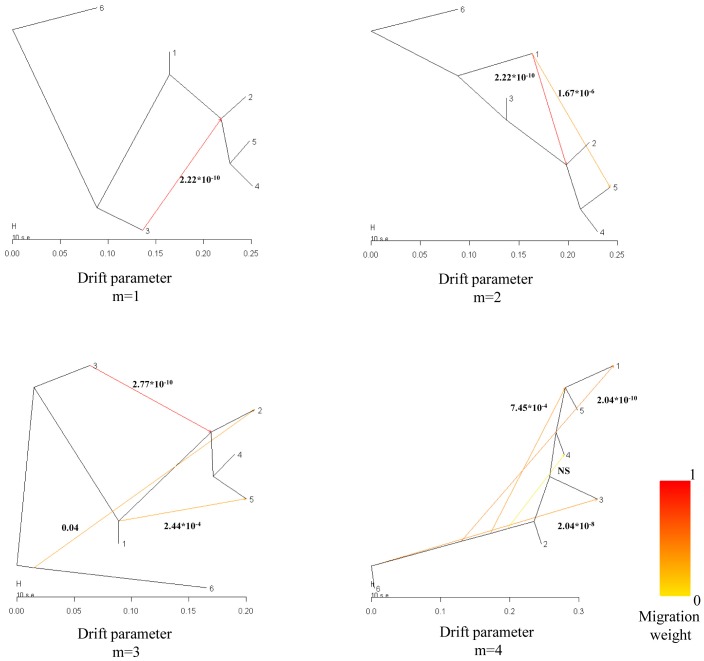
Maximum likelihood trees constructed with TreeMix depicting splits and migration events (m = 1–4) between six *Sus scrofa* populations: 1, Near Eastern wild boar; 2, Mangalitza pigs, 3, Canarian pigs; 4, Iberian pigs; 5, European wild boar; 6, Korean wild boar. Edges, whose color ranges from red to yellow depending on the weight of the migration event (measured as the fraction of alleles coming from the parental population), indicate the direction of gene flow between populations. Probabilities associated with each migration event are represented by *P*-values in bold.

**Figure 3:**
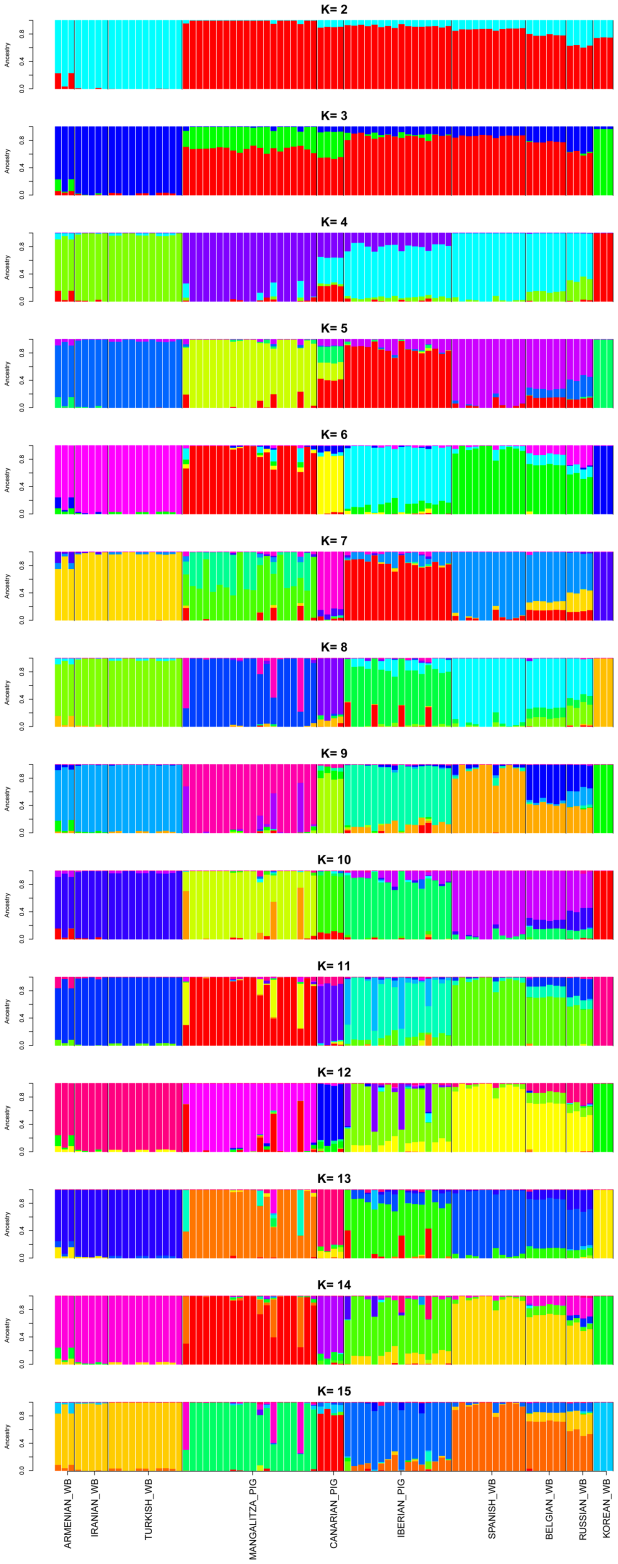
Structure-based estimation of the admixture proportions of 83 individuals belonging to ten *Sus scrofa* populations. The method of Evanno *et al.* [38] indicated that the most likely number of clusters was K = 4, but Admixture analysis and a plot of the log likelihood of each K-value (see Supplementary Information) pointed to K = 5 as the most likely number of clusters. WB = wild boar.

We have examined the 60K SNP data with the TreeMix 1.04 software that allows to infer populations splits and mixtures [19]. Four maximum likelihood trees were built, being very consistent with the genetic relationships delineated above (**Figure 2**). The split between Far Eastern and Western wild boar was evident in all trees, as we chose Korean wild boar as the outgroup. In most trees, there was a clear split between Near Eastern wild boar and the European main cluster (European wild boar and Iberian and Mangalitza pigs), which agrees well with previous reports [1], [5]. The location of Canarian pigs varied remarkably across trees (**Figure 2**). We believe that this feature is the consequence of a distortion in the topology of the tree produced by the mixed European-Far Eastern ancestry and low sample size of this insular breed. Indeed, Canarian swine probably originated through the admixture of indigenous Canarian pigs with Berkshire, Large Black and autochthonous Spanish swine [20]. Berkshire, and to a lesser extent Large Black, are British breeds that have been strongly introgressed with Chinese sows [21], a feature that would explain the presence of Far Eastern alleles in the gene pool of Canarian pigs. The TreeMix analysis also highlighted that Iberian pigs and European wild boar grouped together in three out of four trees supporting the close genetic relationship outlined in our (**Figures 1** and **3**) and previous analyses [5].

Results obtained when modeling from 1 to 4 migration events varied remarkably across trees (**Figure 2**). The violation of one of the implicit assumptions of the migration process modelled by TreeMix, *i.e.* that migration is an instantaneous event that takes place in a short window of time (a premise that is quite unrealistic in the context of pig breeding history), might explain in part this lack of consistency. At m = 1 we found evidence of gene flow between European *Sus scrofa* and Canarian pigs, but its direction was opposite to what was expected (Canarian→European *Sus scrofa*). Indeed, the Canarian breed has a very limited geographic distribution and census (it faced extinction thirty years ago) and there is no reason to believe that it has participated in the foundation of other European breeds. More likely, this result can be explained because of the artificial parental status (*i.e.* basal position in the tree, see previous discussion) of Canarian pigs. We also detected evidence of gene flow between Far Eastern wild boar and Mangalitza pigs when we modeled 3 migration events (**Figure 2**). In spite of the fact that Ciobanu *et al.* [22] have stated that this Balkanian breed was generated several centuries ago by crossing primitive European and Asian pigs, this result is quite contradictory with previous mitochondrial analyses showing that Mangalitza pigs exclusively harbour European haplotypes [23], as well as with data obtained in our study (see Structure results in the next section). Finally, a significant gene flow between Near Eastern and European wild boar was identified when we modeled 2 and 3 migratory events. As we will explain next, this finding was consistent with population structure data obtained with the Structure and Admixture softwares.

Analysis of the data with Structure [24] agreed well with the multidimensional scaling plot displayed at **Figure 1**, by showing that Near Eastern wild boar can be clearly differentiated from European pigs and wild boar (**Figure 3**). At K = 2, we found that samples were distributed in the following two groups: (1) Near Eastern wild boar, (2) European wild boar and pigs and Korean wild boar. This result is not consistent with previous studies [1], [5] showing that the distance between European and Far Eastern *Sus scrofa* is larger than that between European and Near Eastern specimens, a feature that is also reflected in **Figure 1** and that is fully consistent with geography. We believe that this discordant result should be attributed to the low sample size of the Korean group, as previously noted by Goedbloed *et al.* [25] when analysing a European Northwest *Sus scrofa* dataset. As expected, in subsequent analyses with K-values ranging from 3 to 15 the Korean wild boar group was identified as a separate entity from its European and Near Eastern counterparts.

Although the method of Evanno pointed to K = 4 as the most significant K-value, a plot of the log likelihood of K indicated that the true K-value is 5–6 (**Figure S2**). Besides, when we made a second analysis of population structure with the software Admixture (**Figure S3**) we found that the most likely K-value was 5, since Iberian pigs and European wild boar, despite their genetic affinity, were distinguished as belonging to two separate populations. As mentioned by Goedbloed *et al.* [25], the Evanno method tends to underestimate K when genetic differentiation between populations is weak [26]. In the light of these evidences, we believe that the most likely number of clusters is five instead of four *i.e* (1) Near Eastern wild boar, (2) Mangalitza pig, (3) Iberian pig, (4) European wild boar and (5) Korean wild boar, whilst Canarian pigs (but not the Mangalitza ones) had a mixed origin, as previously discussed.

Interestingly, in the Structure analysis (**Figure 3**) the three Iranian, Armenian and Turkish wild boar populations shared a common genetic background that was clearly different from that of European wild boar. This result was very robust for all K-values under consideration. Similar results were obtained with Admixture (**Figure S3**), although at K = 6–10 the Near Eastern cluster was split into two Iranian and Armenian *vs* Turkish subclusters evidencing the existence of population substructure. As a whole, we must conclude that, in contrast with our previous observation [5], the gene pools of Near Eastern and European wild boar can be unequivocally differentiated at the autosomal level. This result agrees well with previous findings [1], [27] indicating the absence of a Near Eastern genetic signature in the mitochondrial gene pool of modern European swine breeds.

An intriguing result was the identification of a Near Eastern genetic signature in the autosomal genomes of Belgian and Russian wild boar, as inferred from the Structure (K = 2–15, **Figure 3**) and Admixture (K = 2–10, **Figure S3**) analyses. This finding was very consistent although its significance is limited by the reduced number of sampled individuals. The TreeMix analysis showed evidence of gene flow from Near Eastern to the European main cluster at m = 2 and 3, but not at other m-values (**Figure 2**). The existence of gene flow between wild boar populations from Russia and Armenia/Iran/Turkey is conceivable because of the close geographic distance between these countries. Assuming this hypothetical scenario, migration of Russian wild boar into Eastern Europe might result in the entry and dispersion of Near Eastern alleles amongst European wild boar populations, likely at low frequencies. However, current evidences, based on extensive mitochondrial analyses, argue strongly against this hypothesis and support much better a scenario of vicariance between European and Near Eastern wild boar populations [1], [27]. Indeed, a mitochondrial analysis specially focused on wild boar from Greece has not revealed any genetic affinity with those of Near East, with the only exception of a few specimens of Samos, which is separated from Anatolia by the 1.6 km Mycale strait [27]. The lack of a Near Eastern signature in the mitochondrial genome of European wild boar does not necessarily imply that it is also absent from the autosomal genome, an issue that should be investigated through the extensive sampling and high throughput genetic analysis of wild boar from Central Asia, Eastern Europe and Near East. Massive sequencing should be used to characterize the variability of Near Eastern wild boar in an unbiased manner and to investigate the presence of Near Eastern alleles in European wild boar and pig breeds.

Finally, we would like to discuss the close genetic relationship we have found between Iberian pigs and European wild boar. The multidimensional scaling plot shown in **Figure 1** evidenced that Iberian pigs are closely related with European wild boar, as previously reported [5], whilst Mangalitza and Canarian pigs happened to be more distantly related and formed independent clusters. The Structure analysis also showed a genetic affinity between Iberian and European wild boar at K = 2–4 (**Figure 3**), whilst at K = 5 both populations appeared differentiated. Calculation of F_ST_ values (**Table 2**) also showed that the level of genetic differentiation between Iberian and Mangalitza pigs (F_ST_ = 0.146) was slightly higher than that between Iberian pigs and European wild boar (F_ST_ = 0.129). Similar results were obtained by Ramírez *et al.* [5] when comparing the autosomal genetic diversity of Iberian pigs and their local wild ancestors, suggesting the existence of a substantial gene flow between both populations after domestication [28]. Mitochondrial analyses have also revealed a tight affinity between Portuguese wild boar and Iberian and Alentejano pigs, which has been interpreted as proof of hybridization events [29]. Some level of pig introgression in Sardinian wild boar has also been detected, suggesting that gene flow between wild and domestic *Sus scrofa* is bidirectional [30]. The analysis of genome-wide SNPs in Northwest European wild boar and domestic pigs from six breeds (Duroc, Landrace, Large White, British Saddleback, Tamworth and Piétrain) revealed that the allele frequency spectrum of analysed wild boar and pigs is remarkaby different, with a high proportion (∼20%) of rare alleles (frequency between 0.005 and 0.03) in the former [25]. These rare alleles might have entered the wild boar gene pool through admixture with pigs from multiple breeds [25]. In our study, however, the proportion of rare alleles in European (4.62%) and Near Eastern (4.16%) wild boar was substantially lower than that observed by Goedbloed *et al.* [25], suggesting that they have not been recently admixed with pigs.

According to Vilà [31], the gene pool of dogs, pigs and cattle could have been significantly enriched through the hybridization with their wild ancestors, being this process particularly important during the early stages of domestication. In this sense, evidence consistent with large-scale backcross between male wild boar and female domestic pigs in East Asia has been recently found [32]. Indeed, unintentional genetic exchanges between pigs and wild boar might have occurred and might still occur. Throughout the ages, pig breeding in Europe has substantially relied on the free ranging and scavenging of swine in the woods [33]. In England, who pioneered much of the technical advances in pig husbandry, the transition to a more intensified production regime, based on pigs confinement in sties, utilization of new feeding sources (*e.g.* peas, beans, dairy waste etc.), exploitation of heterosis through crossbreeding and implementation of selection schemes, did not begin until the end of the 17^th^ century, as a consequence of increasing human population densities and progressive deforestation [33]. Even nowadays, certain breeds from Mediterranean countries, such as the Iberian pigs, are allowed to graze in acorn oak groves reflecting breeding practices (*i.e.* pannage) that in the past were broadly widespread across Europe. Historical data suggest that in the last 9,000 years European pigs and wild boar shared a similar ecological environment [33], thus creating an ample window of opportunity to exchange genetic material. By analysing a very extensive sample of ancient and modern *Sus scrofa* specimens, the Porcine HapMap project is expected to reveal the magnitude, direction and timing of this gene flow as well as to unveil the genetic ancestry of a wide array of European breeds.

## Materials and Methods

### High throughput SNP genotyping

Pig and wild boar genomic DNA obtained in previous studies [5] was employed in the current work. Additional *Sus scrofa* DNA samples were provided by people listed in the Acknowledgments section. These samples have been referenced in diverse publications [34], [35], [36], [37]. The complete dataset is shown at **Table S1**. Nine Turkish and one Iranian samples with low genomic DNA concentration were amplified using the REPLI-g UltraFast Mini Kit (Qiagen) for whole genome amplification. Genotypes were inferred with the Illumina Porcine SNP60 Beadchip following manufacturer instructions. SNP data were filtered by imposing thresholds of 0.95, 0.05, and a *P*-value of 0.00001 for the call rate, minor allele frequency (MAF), and Hardy-Weinberg equilibrium test, respectively. X-chromosome SNP were excluded from the population genetics analyses.

### Data analysis

The multidimensional scaling plot was based on the calculation of genome-wide identity-by-state pairwise distances with the PLINK whole genome association analysis toolset [10]. We used Arlequin 3.5.1.2 software [14] to estimate the partition of molecular variance among and within populations. F_ST_ calculations were carried out with the same software, using 1,000 permutations to infer statistical significance. Average observed and expected heterozygosities were estimated with PLINK v. 1.07 [10]. Multi-locus genotype analysis of population structure was carried out with Structure v. 2.3.3 [24] with the following options: admixture model with 10,000 iterations (the first 2,000 iterations were discarded as burn-in) and considering that allele frequencies are correlated. We considered ten populations: Korean, Armenian, Iranian, Turkish, Russian, Belgian and Spanish wild boar and Mangalitza, Canarian and Iberian pigs. Different values of the number of clusters (K = 2–15) and five separate runs were carried out for each K-value. The most likely number of clusters was inferred with the Evanno method [38] using the web server Structure Harvester [39]. Further, we carried out a parallel analysis with the program Admixture 1.22 [40], which infers population structure from large autosomal SNP genotype datasets and uses a cross-validation procedure allowing to identify the K-value for which the model has best predictive accuracy. The smallest cross-validation error indicates the correct (or most probable) K-value. For the termination criteria we used default parameters since they are already well optimized [40].

The patterns of population splits and mixtures in *Sus scrofa* populations were inferred with TreeMix v. 1.04 [19]. This software delineates the relationships between sampled populations, with a particular emphasis on topology rather than on the timing of demographic events. In the resulting maximum likelihood trees, inferred population splits are represented as nodes and branch lengths are proportional to the amount of genetic drift that populations have undergone. Migration events are modeled for populations that do not fit well the bifurcating tree model, because they have ancestry from multiple parental populations, and they are indicated as edges. The color of the edges reflects the relative weight of migration *i.e.* the fraction of alleles in the descendant population that originated in each parental population (m = 0→1, yellow: small fraction of alleles, red: large fraction). We tested for a range of migration events (m, 1–4), using the Korean population as an outgroup.

## Supporting Information

Table S1 **Sample sizes of the** ***Sus scrofa*** **populations analysed in the current study with the Illumina Porcine SNP60 BeadChip.**
(DOC)

Figure S1 **Multidimensional scaling plot of wild boar and pig populations based on genome-wide identity-by-state pairwise distances.** Korean wild boar was excluded from this analysis to facilitate the visualization of the genetic relationships amongst the remaining populations.
(TIF)

Figure S2 **Estimates of the most likely number of clusters in the Structure analysis derived from the log likelihood associated with each K-value,** ***i.e.*** **L(K) mean and standard deviation (blue points), and the second order rate of change of the likelihood,** ***i.e.*** **delta K (red points).** When K is approaching to its true value, L(K) reaches stability or decreases moderately.
(TIF)

Figure S3 **Bar-plot of Admixture results and cross-validation error for each K- value.** The lowest CV-error indicates the most likely K-value (K = 5).
(PPT)
