# Supplementary material for: A High Throughput Genotyping Approach Reveals Distinctive Autosomal Genetic Signatures for European and Near Eastern Wild Boar
Source: PLoS One. 2013 Feb 27;8(2):e55891. doi: 10.1371/journal.pone.0055891 (PMC3584081; doi:10.1371/journal.pone.0055891)
Supplement: Table S1 — Sample sizes of the Sus scrofa populations analysed in the current study with the Illumina Porcine SNP60 BeadChip. (DOC) [file pone.0055891.s001.doc]

**Supporting Information Table 1**

| **Populations** | **Country** | **N** |
| --- | --- | --- |
| European wild boar | Russia | 4 |
|  | Belgium | 6 |
|  | Spain | 11 |
|  | **TOTAL** | 21 |
| Near Eastern wild boar | Iran | 5 |
|  | Turkey | 11 |
|  | Armenia | 3 |
|  | **TOTAL** | 19 |
| Far Eastern wild boar | Korea | 3 |
| European pigs | Canarian | 4 |
|  | Iberian | 16 |
|  | Mangalitza | 20 |
|  | **TOTAL** | 40 |
| **Total number of samples** | **ALL** | **83** |
