# Supplementary figures and images for: A High Throughput Genotyping Approach Reveals Distinctive Autosomal Genetic Signatures for European and Near Eastern Wild Boar
Source: PLoS One. 2013 Feb 27;8(2):e55891. doi: 10.1371/journal.pone.0055891 (PMC3584081; doi:10.1371/journal.pone.0055891)

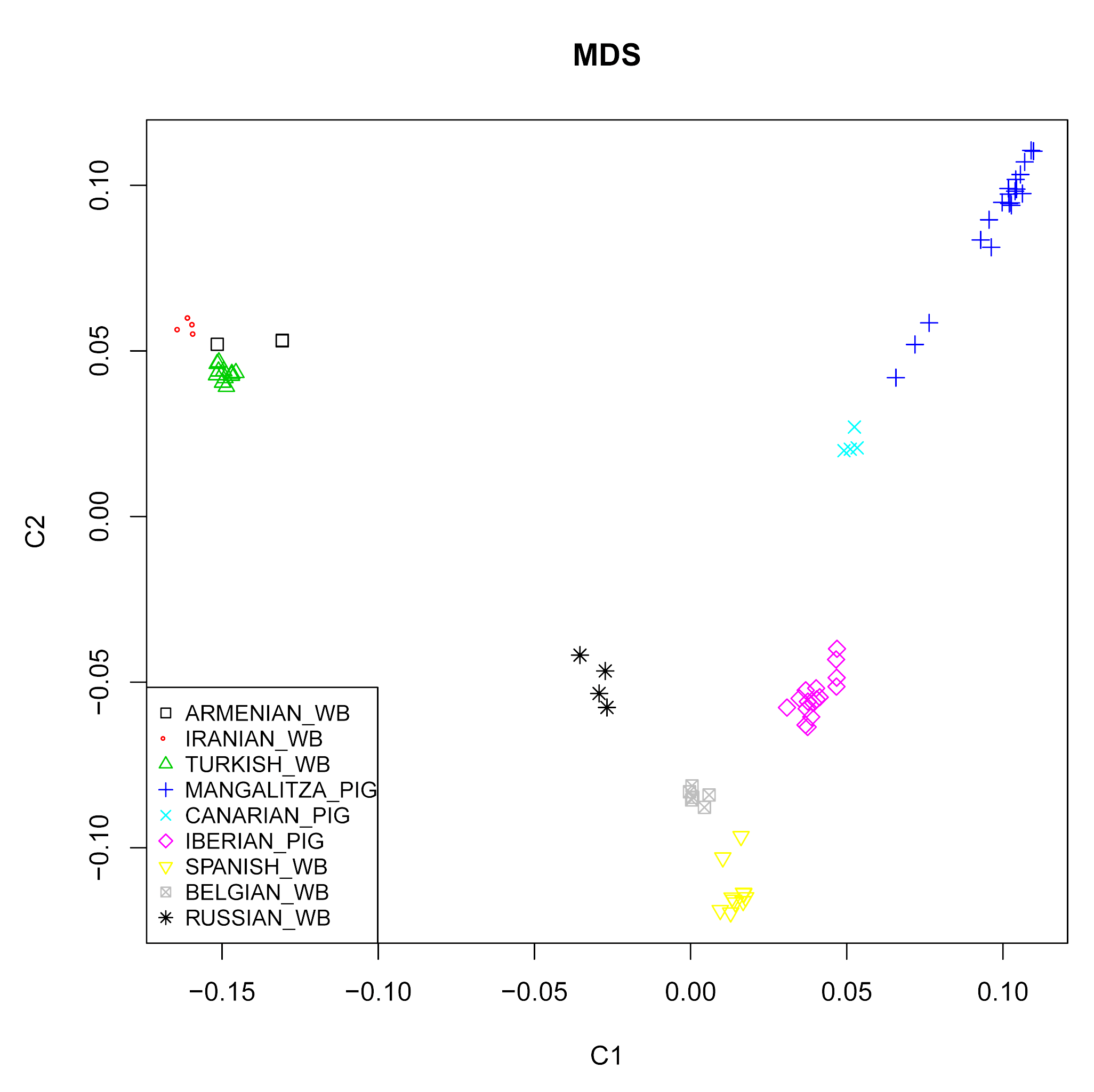

Supplement: Figure S1 — Multidimensional scaling plot of wild boar and pig populations based on genome-wide identity-by-state pairwise distances. Korean wild boar was excluded from this analysis to facilitate the visualization of the genetic relationships amongst the remaining populations. (TIF) [file pone.0055891.s002.tif]

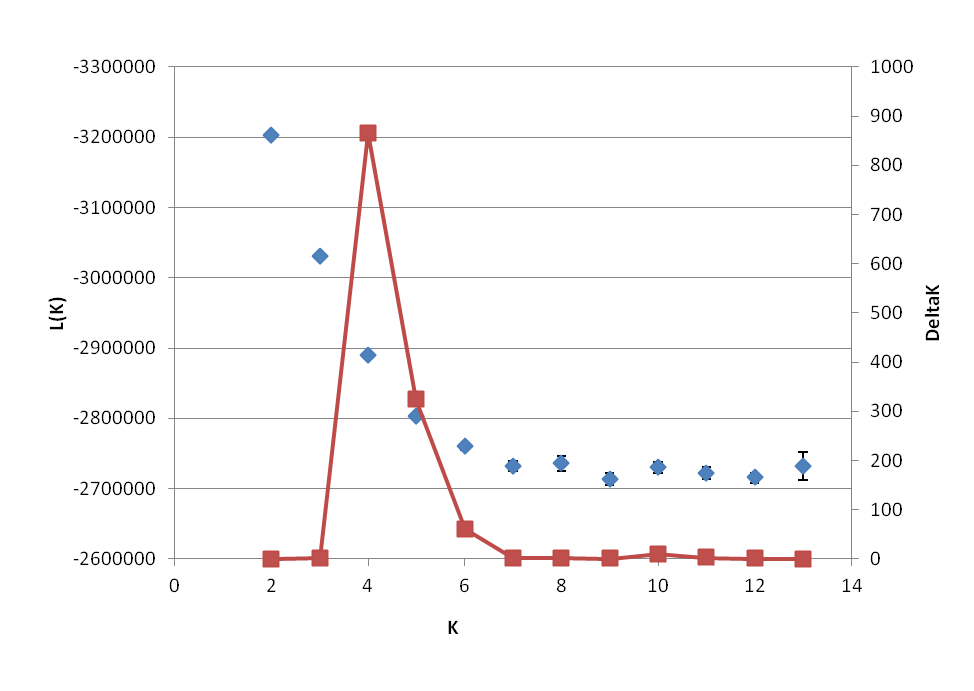

Supplement: Figure S2 — Estimates of the most likely number of clusters in the Structure analysis derived from the log likelihood associated with each K-value, i.e. L(K) mean and standard deviation (blue points), and the second order rate of change of the likelihood, i.e. delta K (red points). When K is approaching to its true value, L(K) reaches stability or decreases moderately. (TIF) [file pone.0055891.s003.tif]

## Slide 1
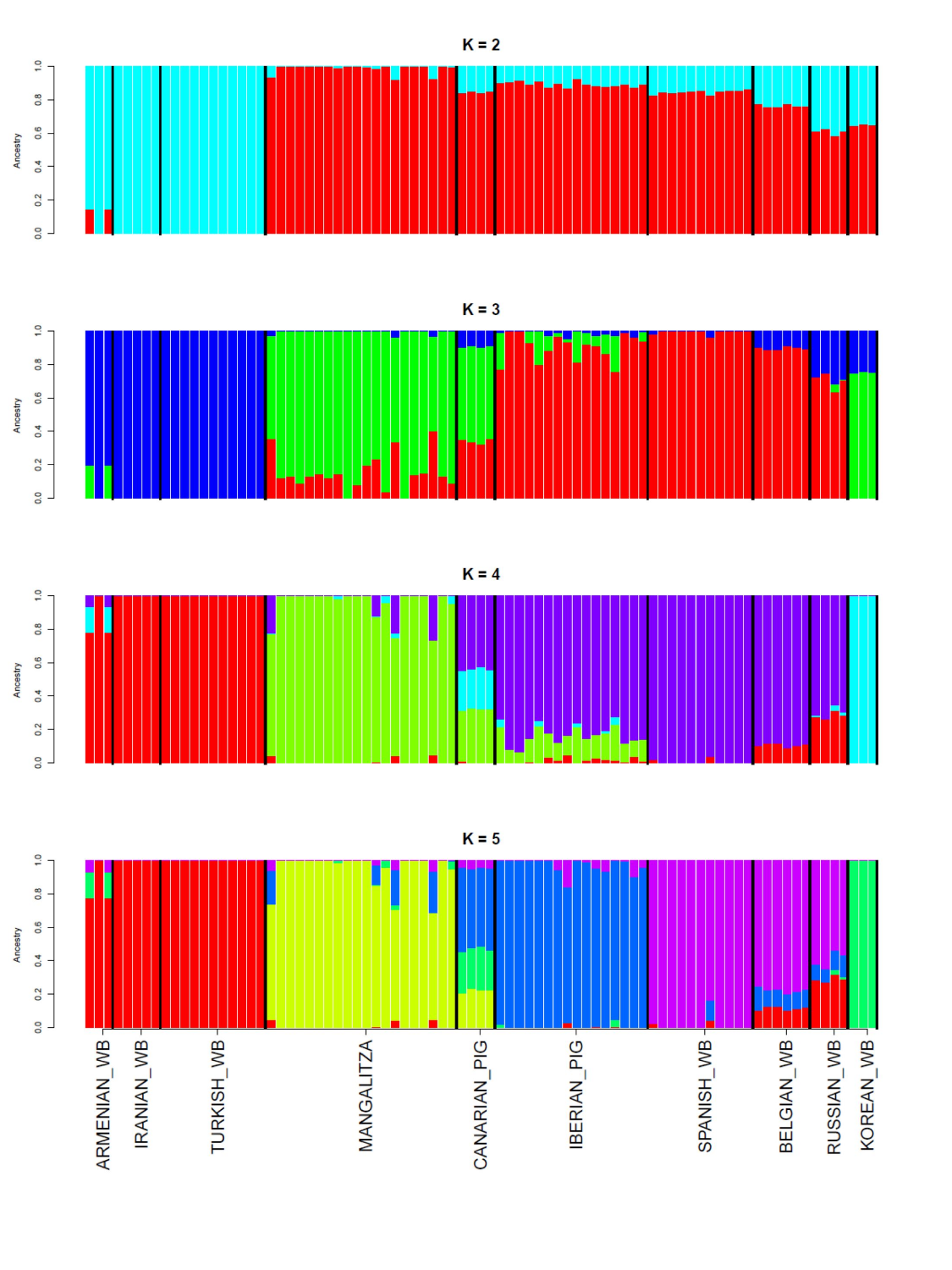

## Slide 2
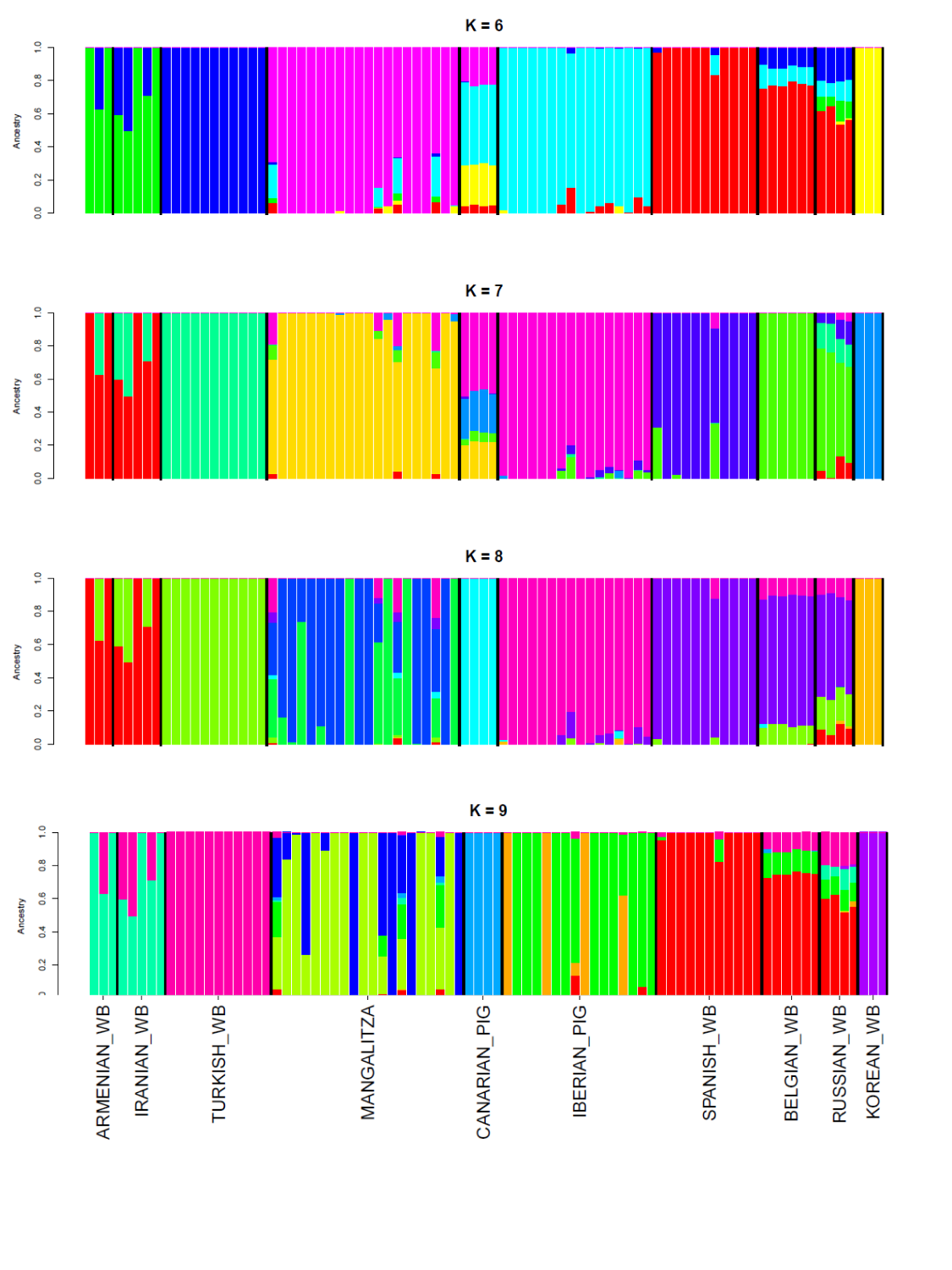

## Slide 3
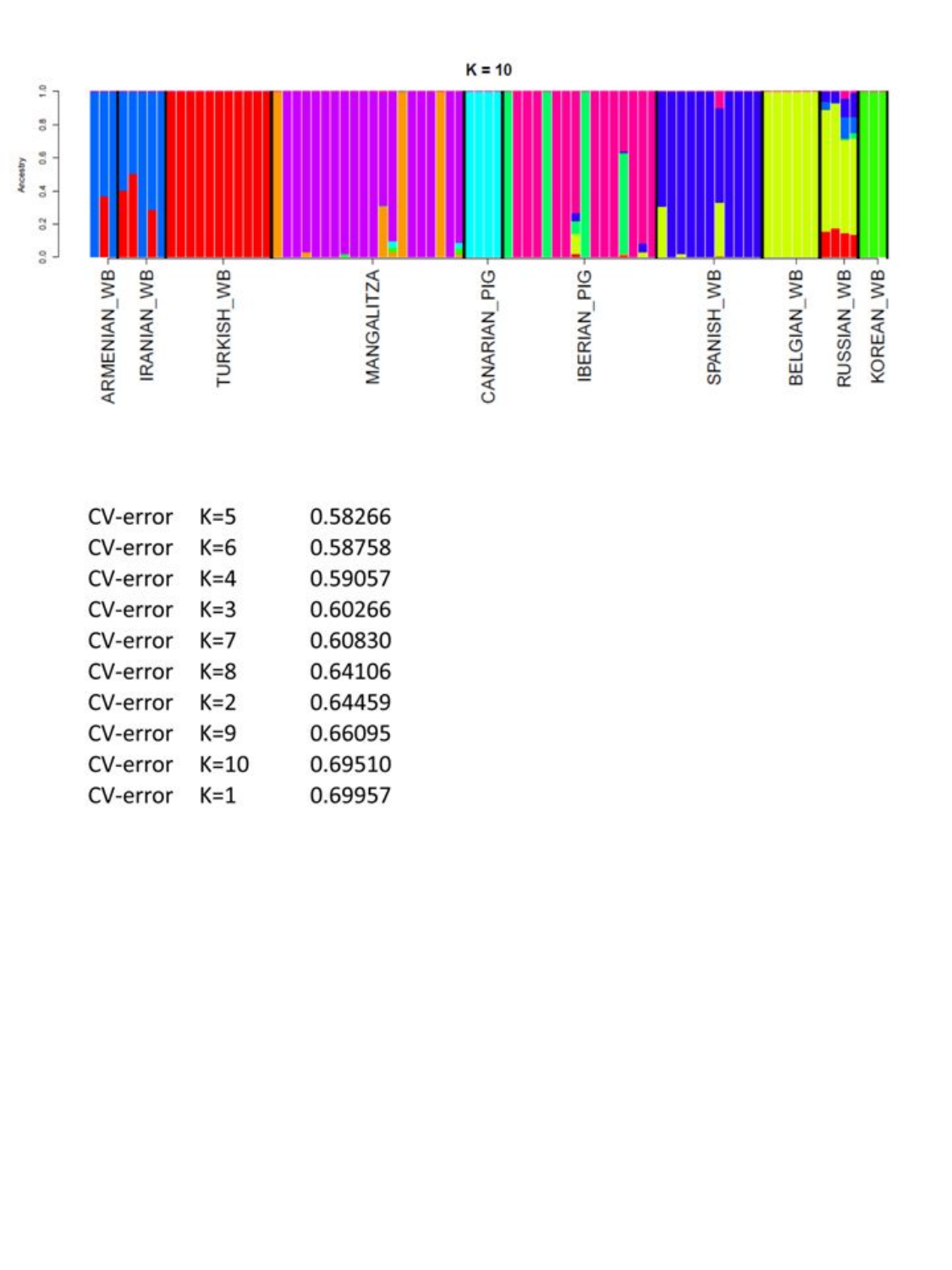

Supplement: Figure S3 — Bar-plot of Admixture results and cross-validation error for each K- value. The lowest CV-error indicates the most likely K-value (K = 5). (PPT) [file pone.0055891.s004.ppt]
